# APSec1.0: Innovative Security Protocol Design with Formal Security Analysis for the Artificial Pancreas System

**DOI:** 10.3390/s23125501

**Published:** 2023-06-11

**Authors:** Jiyoon Kim, Jongmin Oh, Daehyeon Son, Hoseok Kwon, Philip Virgil Astillo, Ilsun You

**Affiliations:** 1 School of Computer Sciences, Gyeonsang National University, Jinju-si 52828, Republic of Korea; jykim92@gnu.ac.kr; 2 Department of Financial Information Security, Kookmin University, Seoul-si 02707, Republic of Korea; dhwhdals2287@kookmin.ac.kr (J.O.); sondh97@kookmin.ac.kr (D.S.); hoseok1997@kookmin.ac.kr (H.K.); 3 Department of Computer Engineering, University of San Carlos, Cebu City 6000, Philippines; pvbastillo@usc.edu.ph

**Keywords:** artificial pancreas system (APS), security protocol, formal verification

## Abstract

The Medical Internet-of-Things (MIoT) has developed revolutionary ways of delivering medical care to patients. An example system, showing increasing demand, is the artificial pancreas system that offers convenience and reliable support care to patients with Type 1 Diabetes. Despite the apparent benefits, the system cannot escape potential cyber threats that may worsen a patient’s condition. The security risks need immediate attention to ensure the privacy of the patient and preserve safe functionality. Motivated by this, we proposed a security protocol for the APS environment wherein support to essential security requirements is guaranteed, the security context negotiation is resource-friendly, and the protocol is resilient to emergencies. Accordingly, the security requirements and correctness of the design protocol were formally verified using BAN logic and AVISPA, and proved its feasibility through the emulation of APS in a controlled environment using commercial off-the-shelf devices. Moreover, the results of our performance analysis indicate that the proposed protocol is more efficient than the other existing works and standards.

## 1. Introduction

**Figure 1:**
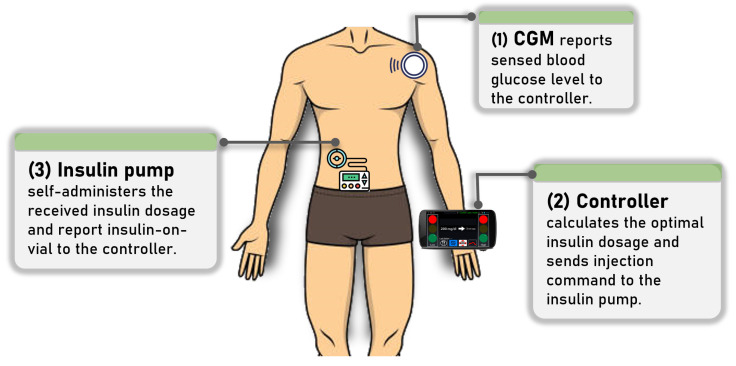
An exemplary illustration of the Artificial Pancreas System.

Artificial pancreas system (APS) is a closed-loop insulin injection system, which can be classified as an Implantable Medical Device (IMD). It continuously monitors the glucose level of a patient with Type 1 Diabetes (T1D) and assists them with the insulin injection process. These systems will improve the glycemic control of the patient and reduce the risk of hypoglycemia and hyperglycemia, ultimately enhancing the quality of life of patients, especially those that suffer from T1D and other insulin-dependent conditions [1,2]. Traditional insulin injection requires two steps. First, the patient checks the glucose level through blood drop. After that, the medical attendant manually injects physician-prescribed insulin dosage if the level goes beyond the normal range. Meanwhile, state-of-the-art APS automates these procedures with two Internet-of-Things (IoT) devices and one controller device, also serving as the IoT gateway. One of the IoT devices continuously monitors, at a configured interval, the glucose level and forwards it to the controller. Subsequently, the controller, through its visualization feature, presents the observed level, as well as calculates the appropriate insulin dosage, which is sent as part of the command message to the insulin-injecting IoT device. An exemplary illustration of the APS is shown in Figure 1.

The demand for APS has lately been increasing due to the substantial growth of pancreatic ailment and failure cases. According to a report by IMARC Group, the global device market of APS has reached USD 1,949 million in 2021. The market is expected to reach USD 4,122 million by 2027. This represents a compound annual growth rate (CAGR) of 13.4% during the period from 2022 to 2020 [3]. Market Research Future also projected that in the period between 2022 and 2023, the market will generate sales of USD 341.08 million and an increase in CAGR of 18.0% [4].

With the increasing demand for not only APS but IMDs in general, new security risks also arise that need attention to ensure the privacy of patients and preserve safe functionality. In recent years, several studies have highlighted the vulnerability of IMDs, such as APS, to a variety of security threats [5,6]. The consequences of a successful attack on IMDs can range from device malfunction to life-threatening situations. Just as the previous research indicates, the usage of APS is increasing fast, along with its impact on society. Recently highlighted risks associated with APS include the unauthorized access to the devices from attacker, intercepting sensitive medical data, and even taking control of the devices themselves.

Although APS has apparently transformed classical medical care practices to be more convenient, living with elevated effectiveness for maintaining glucose to normal level, there are still several key issues in terms of cybersecurity that needs immediate resolution [7,8].

The basic structure of APS is composed of three resource-constrained devices, which communicate with each other wirelessly at certain intervals. Hence, considering the nature of such an environment, suitable secure communication protocol with low energy consumption is vital. Accordingly, these devices can authenticate, verify, and securely exchange message with each other. With such a motive, this paper has examined existing protocols for IMDs and IoT devices. For example, Bluetooth is the most widely adopted protocol in IoT ecosystem. However, its security level is not suitable for deployment in IMDs, especially in APS. Other existing protocols also had their own drawbacks if employed in the APS environment, and, more importantly, all of them are not resilient in case of emergency situations. Thus, this paper proposes a security protocol, dedicated to the APS, while considering the three vital criteria, being secure, lightweight, and resilient. In addition, the proposed protocol is also aligned with the security considerations from guidance documents for medical device of the US Food and Drug Administration (FDA) [9,10].

The main contributions of this paper are summarized as follows:

- Designing an innovative security protocol to establish tripartite authentication and construct a secure channel between the involved devices of the APS.
- Formally verifying satisfaction with vital security services as well as the correctness of the proposed protocol.
- Comparing the protocol with existing IoT-intended protocol and well-known security standards in terms of security requirements, computation, and communication overheads.
- Demonstrating the feasibility of the protocol through emulation in a controlled environment using commercial off-the-shelf devices.

The rest of the paper is organized as follows. Section 2 discusses an overview of an IMD system. Section 3 describes the proposed protocol. Section 4 presents the formal security analysis and results from BAN logic and AVISPA. Section 5 presents the experimental environment and implementation. Finally, Section 6 concludes the paper.

## 2. Related Works

Insulin is an important hormone that regulates blood glucose levels in the body. However, for individuals with insulin receptor deficiency, insulin resistance, or insulin secretion deficiency, insulin injections are necessary. It is one of the most common treatments for diabetes; however, insulin injections can be inconvenient in daily life. Patients must inject themselves at regular intervals and continuously monitor their blood sugar levels and insulin doses. This can be a significant source of stress for many diabetes patients.

Recently, the concept of an APS [11,12] has emerged as a solution to these problems. APS is a device that automatically performs continuous blood glucose monitoring and insulin administration. This replaces the role of the pancreas in secreting insulin to regulate blood glucose levels. APS enhances the convenience and safety of daily life of blood glucose control in diabetic patients by automating the process. In addition, it improves the accuracy of blood glucose regulation, reducing the incidence of complications associated with diabetes.

However, there are also several challenges associated with the development and implementation of an APS. One of the most critical issues is ensuring the security and privacy of patient information. Since APS uses sensitive personal information, such as medical records and blood glucose levels, it is essential to establish robust information security protocols to prevent unauthorized access, data breaches, and cyberattacks. Therefore, information security measures (e.g., security protocol) should be designed to ensure that patient data are protected and that patients can use APS with confidence.

Unfortunately, it is difficult to find significant existing research on security protocols specifically designed for APS. Instead, we expand our scope and review existing research on security protocols for IMD. The following existing studies on IMD-based security protocols were proposed in similar environments to APS. However, it is difficult to say that they fully support APS due to the various differences between APS and IMD, such as requirements, structure, device, and performance.

Wu et al.’s protocol [13] is a novel fine-grained IMD access control scheme based on a proxy device, such as the patient’s smartphone, which will delegate the heavy access control computations for the IMDs. The communications between the proxy and the IMD programmer are conducted through an audio cable to mitigate potential wireless attacks. The ciphertext-policy attribute-based encryption (CP-ABE) is employed to enforce fine-grained access control over the qualifications of the programmer operator. The IMD holds a unique identifier and master key, used only in the initial pairing process with the proxy. All operators with Public Key Infrastructure (PKI) parameters used in CP-ABE must first be registered with the Central Health Authority (CHA), which creates and manages secret keys used in communication. The operator obtains information by inputting or reading through the smart card and communicates with other devices using the programmer. The proxy connects with the programmer to control access to the IMD. In spite of this protocol’s novelty, its need for an audio cable connection between the proxy and the IMD programmer may be inconvenient or impractical in certain situations. Moreover, compared to traditional PKI algorithms, CP-ABE results in relatively excessive computation overhead while not widely being applied.

Chi et al.’s protocol [14] e-SAFE aims to provide secure, efficient, and forensics-enabled access to IMDs. The system uses a smartphone as a proxy to undertake most of the security-related tasks and establishes a trusted channel between the IMD and the smartphone by sharing a secret key derived from a master key that is physically inaccessible to attackers. The doctor responsible for all operations on the patient’s IMDs is authenticated, and access privileges are authorized through widely used SMS/email services. Furthermore, this protocol uses a compression-based encryption mechanism, PKI, and other cryptographic protocols. The IMD connects with the patient’s smartphone via Bluetooth and the programmer of the doctor via the wireless medium. The patient’s smartphone communicates with the IMD via Bluetooth and the programmer of the doctor via wireless. On the one hand, the protocol can provide secure access to IMDs, reducing the risk of malicious attacks. The authentication of doctors and authorization of access privileges ensure that only authorized personnel can access IMDs. The forensics-enabled feature allows for post-event analysis in case of any security breaches. On the other hand, using SMS/email services for authorization may not be secure enough for some users.

The HAT protocol [15] is a solution that provides fine-grained and dynamic access control for the next generation of IMDs while offering full control and transparency to the patient. This protocol can provide a secure key exchange that relies on a hash-based access token between personal devices and the IMD. The protocol involves three parties, the IMD, the patient’s personal device, and a security manager. The security manager is an external device under the patient’s control that issues access tokens to securely establish session keys between personal devices and the IMD. The security manager role can be assigned to a new external device at any time, revoking the former security manager. This protocol ensures that only authorized devices can communicate with an IMD, providing fine-grained and dynamic access control for patients while maintaining their privacy and security. The advantage of this protocol is that it allows for easy initialization or connection of new devices, and multiple personal devices can communicate with IMD simultaneously.

HAT exchanges session key between IMD and personal device through Elliptic Curve Diffie–Hellman Ephemeral (ECDHE) under the supervision of a security manager. Subsequently, the security manager is excluded from communication, and IMD and personal device communicate directly. However, in the APS environment, there are two medical devices (CGM, Insulin Pump) that need to be connected to the patient’s personal device (Controller). At the same time, an emergency phase is required to protect the patient from accidental incidents when the Controller becomes inoperable. HAT is not entirely suitable for the requirements of APS presented in Section 3.

The Bluetooth LE (BLE) protocol [16] is one of the low-power communication technologies designed for wireless devices. It enables low-power communication between devices, characterized by very low power consumption and short transmission distances. This allows for long operation times with small batteries. Additionally, it provides faster connection speeds and a larger range than the traditional Bluetooth protocol while reducing costs by using inexpensive wireless modules. However, considering to apply it for the APS, there are some disadvantages, such as two-party protocol, low data transfer rates and limited processing capabilities, complex implementation processes, and a lack of compatibility with the traditional Bluetooth protocol. More importantly, its key exchange protocol is not authenticated, thus being vulnerable to Man-in-The-Middle (MiTM) attacks.

The TLS 1.3 protocol [17,18] offers higher security than previous versions, reduces connection setup time, and enhances privacy protection. It addresses security flaws and vulnerabilities in earlier versions and improves resilience to new attack techniques. Additionally, the handshake process has been improved to speed up session establishment, and various features have been added to enhance privacy protection. However, despite its popularity and advanced architecture, it is relatively complex, a two-party protocol, and requires heavy computation overhead, which is thus clearly not proper for the APS.

## 3. Proposed Protocol

APS can relieve the inconvenience for patients who have to manage their health conditions due to various diseases continuously. For example, insulin pumps are used for patients who require multiple insulin injections, such as Type 1 Diabetes and gestational diabetes. Conventional insulin injection therapy is a method that can control a patient’s blood sugar effectively. Still, it is accompanied by the inconvenience (pain, fear, etc.) of an injection needle for each administration. Insulin pumps can relieve not only the inconvenience of insulin injection therapy but also prevent missed or misuse of medications.

Meanwhile, APS collects the patient’s condition to provide various functions to the patient and repeats the process of injecting medicine based on this. In other words, APS can collect and judge sensitive information, such as the patient’s health status. Such sensitive information and the operation of devices based on it can become a target for malicious attackers to damage users’ financial and physical values. Therefore, the communication of the APS device must be sufficiently protected from malicious attackers. In this chapter, we design a security protocol that supports authentication and key negotiation to protect patient-sensitive information from malicious attackers in communication between APS devices. The terms used in this paper and their meanings are shown in Table 1.

Before delving into designing security protocols, it is necessary to analyze the target environment and define security requirements and assumptions. A description of the target environment is provided in the Introduction, and in this chapter we define security requirements and assumptions and design security protocols.

### 3.1. Security Requirement

Security requirements refer to the demands that an organization or system must fulfill to operate securely and to protect the information, assets, and users’ personal data. While there can be various forms of security requirements, defining appropriate security requirements is desirable. Therefore, the process of deriving security requirements through the analysis of the target environment is crucial in designing security protocols.

APS is a medical device that delivers insulin automatically to diabetes patients. Therefore, to protect the patient’s health and privacy, it is important to design the system with security as the top priority. The following are some of the security requirements for APS:

**Mutual Authentication.** Mutual authentication plays a crucial role in establishing a trusted relationship by verifying and confirming the trustworthiness of each other. Through mutual authentication, it is possible to verify the trustworthiness of communication counterparts and set up access controls for security and privacy protection. Since the APS environment deals with sensitive data such as patient health information, it is necessary to provide access control through mutual authentication to protect it. Mutual authentication can be implemented using various methods, such as encryption, digital signatures, certificates, tokens, and other mechanisms.

**Confidentiality and Integrity.** Confidentiality and integrity are two key principles of information security. Confidentiality refers to the protection of sensitive information so that it is only accessible to authorized users or systems. In other words, sensitive information should be disclosed only to users with appropriate permissions. Integrity means that information remains unchanged and in its original state, as authorized by users or systems. APS that transmits sensitive information, such as users’ health information, should protect the information from unauthorized alteration or damage through confidentiality and integrity. Confidentiality and integrity are complementary principles in information security. Therefore, a comprehensive information security strategy should consider both confidentiality and integrity and implement them rigorously to ensure the security of information.

**Secure Key Exchange.** Secure key exchange is an important concept in computer security and cryptography, used to establish shared secret keys for secure encrypted communication. A key exchange is the process of exchanging confidential information between two entities to generate shared secret keys for use in communication. There are various methods for key exchange, and the choice of method should be based on the requirements of the communication system and security policies. Secure key exchange is a critical step in ensuring the confidentiality, integrity, authentication, and other security properties of communication and should be carefully selected and implemented.

**Perfect Forward Secrecy** Perfect Forward Secrecy (PFS) is a property of cryptographic protocols that ensures that if an attacker manages to obtain the secret key of a particular session, they will not be able to use it to decrypt past or future sessions. In other words, PFS ensures that the secrecy of past and future communications remains intact even if the secret key of a particular session is compromised. PFS is typically achieved through the use of temporary session keys that are generated for each session and are not derived from a long-term secret key. These session keys are used to encrypt and decrypt data for the duration of the session, and they are discarded once the session is completed. As a result, even if an attacker manages to obtain the session key for a particular session, they will not be able to use it to decrypt any past or future sessions.

**Privacy.** Privacy protection refers to the safeguarding of an individual’s personal information and activities. It involves various means and methods to ensure that personal information is not collected, used, or disclosed without authorization. Privacy protection respects the rights and freedoms of individuals and includes principles and technologies for appropriately handling and securing personal information. This can be achieved through techniques and procedures, such as secure processing of personal information, encryption, access controls, user consent, and data integrity. Privacy protection aims to enhance trust and security in handling personal information while respecting and safeguarding an individual’s privacy. In an APS environment, security protocols that ensure privacy can safeguard against attacks involving the recognition, identification, or tracking of the user’s APS device by potential attackers.

**Emergency Phase.** APS is a critical system that directly impacts the health of patients, and it should be prepared for various disruptions caused by different factors. The proposed protocol is designed to exchange emergency session keys to overcome certain disruptions caused by control device failures in emergency situations.

### 3.2. Assumptions

Assumptions refer to the essential elements that should be considered for a security protocol to meet the security requirements in the target environment. The assumptions for the proposed protocol are as follows:

1. The security protocol proposed in this paper is designed based on the APS.
2. The security protocol proposed in this paper is the communication of three participants, CGM, Controller (CONT), and Insulin Pump (IP).
3. The protocol proposed in this paper communicates through an open wireless channel.
4. CGM and IP share their passcode safely with CONT.
5. Emergency situation means a situation where the CONT cannot communicate normally with CGM or IP for any reason.

### 3.3. Proposed Protocol

**Figure 2:**
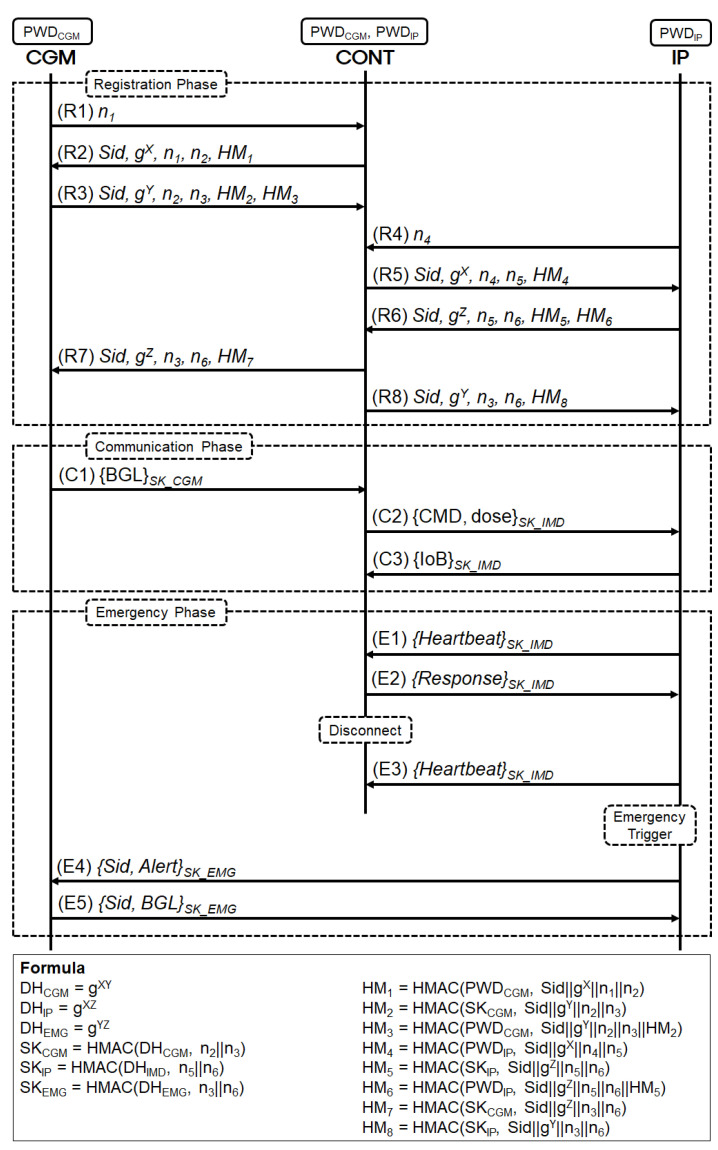
Proposed protocol for secure communication of APS.

The proposed protocol consists of three phases, (1) the registration phase, (2) the communication phase, and (3) the emergency phase. The design of the proposed protocol is shown in Figure 2 and detailed descriptions are below.

#### 3.3.1. Registration Phase

In the first step, CGM and IP must authenticate to establish a secure channel with the controller before transmitting patient information. The Registration phase in Figure 2 shows the certification process between IP and CONT, as well as CGM and CONT. The detailed descriptions of the Registration phase are as follows. Before starting the procedure, CGM and IP share their passcode ($PWD_{CGM}$ and $PWD_{IP}$) with CONT through a secure channel. Furthermore, all the communication participants generate ECDH key pairs. In this paper, we assume the ECDHE (ECDH Ephemeral) option that can use ephemeral keys and support perfect forward secrecy. The key generation and exchange process of the ECDHE option is shown in Table 2.

(R1) After sharing passcodes and generating ECDHE key pairs, CGM generates and transmits a random nonce ($n_{1}$) to trigger a connection with CONT.
(R2) Upon receiving the message sent by CGM, CONT sends a response message that includes a session identifier (Sid) indicating the current session, the ECDHE public key ($g^{X}$) generated in (R1), a fresh nonce ($n_{1}$) received from CGM, and a newly generated random number ($n_{2}$) is included in the message. The message in this step can be protected by the message authentication code ($HM_{1}$) generated using CGM’s passcode ($PWD_{CGM}$) by computing $HM_{1}=HMAC(PWD_{CGM},Sid||g^{X}\left|\right|n_{1}\left|\right|n_{2})$.
(R3) CGM first checks HM1 in the message the CONT sends and, if valid, creates a new random number (n3). After that, CGM calculates the ECDHE secret key ($g^{X}Y$) using the CONT’s ECDHE public key ($g^{X}$) and its own ECDHE private key (*Y*). Furthermore, the session key ($SK_{CGM}$) will be calculated with the ECDHE secret key ($g^{XY}$) and random numbers ($n_{2}$ and $n_{3}$) of CGM and CONT. To protect the messages in this step, CGM includes message authentication codes ($HM_{2}$ and $HM_{3}$) in the outgoing message, which are protected by $SK_{CGM}$ and $PWD_{CGM}$, respectively. Upon receiving this, CONT checks $HM_{3}$ first, and if it is valid, it calculates the ECDHE session key ($g^{XY}$) by combining the CGM’s ECDHE public key ($g^{Y}$) and random numbers. Finally, $HM_{2}$ is checked with the ECDHE session key to verify that the same key was exchanged securely with the CGM. $HM_{2}$ and $HM_{3}$ is calculated as $HM_{2}=HMAC(SK_{CGM},Sid||g^{Y}||n_{2}||n_{3})$, $HM_{3}=HMAC(PWD_{CGM},Sid||g^{Y}||n_{2}||n_{3}||HM_{2})$, separately.
(R4)–(R6) In steps (R4) to (R6), steps (R1) to (R3) described above are equally performed between the IP and the CONT. Through steps (R4) to (R6), IP and CONT satisfy mutual authentication and securely exchange the ECDHE session key ($g^{YZ}$). The message authentication codes $HM_{4}$, HM5, and $HM_{6}$ are calculated as $HM_{4}=HMAC(PWD_{IP},Sid||g^{X}\left|\right|n_{4}\left|\right|n_{5})$, $HM_{5}=HMAC(SK_{IP},Sid||g^{Z}\left|\right|n_{5}\left|\right|n_{6})$, $HM_{6}=HMAC(PWD_{IP},Sid||g^{Z}\left|\right|n_{5}\left|\right|n_{6}||HM_{5})$, separately.
(R7) When the CONT completes authentication with CGM and IP, it sends the IP’s ECDHE public key to CGM. The transmission message is protected by the included message authentication code ($HM_{7}$) and the ECDHE Session Key ($g^{XY}$) between CGM and CONT. $HM_{7}$ is calculated as $HM_{7}=HMAC(SK_{CGM},Sid||g^{Z}\left|\right|n_{3}\left|\right|n_{6})$.
(R8) Contrary to the above, CONT sends the CGM’s ECDHE public key to IP. The message in this step also is protected by the message authentication code ($HM_{8}$) and the ECDHE Session Key ($g^{YZ}$) between IP and CONT. $HM_{7}$ is calculated as $HM_{8}=HMAC(SK_{IP},Sid||g^{Y}\left|\right|n_{3}\left|\right|n_{6})$.

#### 3.3.2. Communication Phase

If CGM and IP successfully authenticate with CONT, they can transmit the patient’s medical information through a secure channel. The Communication phase in Figure 2 represents the process of measuring patient information with a medical device and transferring it to a suitable device. In this case, CGM measures the patient’s blood glucose level (BGL), and CONT converts it into appropriate command and dose messages. The converted message directs IP to inject the appropriate insulin into the patient and return the Insulin on Board (IoB) information to CONT.

IoB refers to the amount of active rapid-acting insulin currently present in the user’s body. This is important as rapid-acting insulin can remain active in the body for up to 3–5 h, and maintaining an appropriate level of IoB is crucial for proper blood sugar control. Checking IoB is included in the Communication phase of this paper, along with blood glucose analysis and insulin administration. The detailed procedure of the Communication phase is outlined below.

(C1) If CGM and IP have successfully generated session keys ($SK_{CGM}$, $SK_{IP}$) with the CONT during the Registration phase, the Communication phase can be initiated. CGM is a device that monitors the user’s blood glucose, and the blood glucose information should be treated as sensitive personal information. Therefore, CGM encrypts the Blood Glucose Level (BGL) using the session key ($SK_{CGM}$) agreed upon with CONT and sends it to CONT.
(C2) Once CONT receives the BGL from CGM, it calculates the appropriate insulin dosage for the user’s blood glucose and generates a command for it. Then, it encrypts the generated command and insulin dosage (CMD, dose) using the session key ($SK_{IP}$) agreed upon with IP and sends it to IP.
(C3) IP administers insulin to the user based on the received command (CMD) and insulin dosage (dose) from the Controller. After administering the insulin, IP generates a message that includes information such as the Insulin on Board (IoB) and the remaining insulin in the device. The message is then encrypted using the session key ($SK_{IP}$) and sent to CONT for reporting.

#### 3.3.3. Emergency Phase

APS is an important device that improves the convenience of patients with diabetes, but it can experience mechanical or communication problems due to various reasons. A malfunction in APS implies not only a device failure but also an immediate health problem for the user. Therefore, an Emergency phase is proposed in which CGM and IP exchange messages to administer insulin by themselves, assuming CONT cannot function properly. Of course, it would be safe for the user to prepare an emergency insulin and blood glucose meter in preparation for such a situation.

(E1)–(E3) IP periodically encrypts and sends Heartbeat messages to CONT to check if it is functioning properly. If CONT is usually working, it can send a response message to IP when it receives the Heartbeat message. On the other hand, when the communication between CONT and IP is disconnected due to mechanical or communication failure, CONT cannot deliver the response message for the Heartbeat message. At this point, IP detects Emergency Trigger and starts the Emergency phase.
(E4) To notify CGM that the Emergency phase has begun, IP sends the session identifier (Sid) used in Registration and the warning message (Alert) to CGM. At this stage, IP and CGM calculate the emergency session key $(SK_{EMG}$). The emergency session key $(SK_{EMG}$) is generated using the mutual ECDHE public keys transmitted by the CONT in steps (R7) and (R8) of the Registration phase, their own ECDHE private key, and nonce values.
(E5) CGM generates an Emergency Request message that includes the session identifier (Sid) and the user’s blood glucose level (BGL), which is encrypted with the emergency session key ($SK_{EMG}$), and sends it to IP. IP administers the required insulin dose based on the received BGL and a predetermined critical value, and then encrypts the Emergency Response message with the emergency session key ($SK_{EMG}$) that includes the session identifier (Sid) and sends it to CGM.

## 4. Formal Verification

In this section, we analyze the proposed security protocol through well-known formal security analysis tools, BAN logic [19] and AVISPA [20]. Both have been used by many researchers to prove the security of their approaches. Security protocols can exhibit unintended behavior due to design flaws, the ambiguity of messages, or complex interactions. Therefore, verification is required to ensure the security of the proposed protocol. BAN logic was proposed by M. Burrows, M. Abadi, and R.M. Needham in 1989, and AVISPA was proposed by A. Armando’s research team in 2005. To prove security through analysis tools, it is necessary to convert the proposed security protocol into an appropriate model for each tool. The proposed security protocol consists of the Registration phase, the Communication phase, and the Emergency phase, and the converted models and verification process for each tool are shown in the subsection below.

### 4.1. BAN Logic

BAN logic [19] sequentially goes through the process of idealization, assumptions, goals, and derivation to verify the security of a protocol. The notations and rules of BAN logic used in the above process are shown in Table 3 and Table 4. First, in the idealization stage, unnecessary elements are removed from BAN logic to model the security of the proposed protocol. In the assumption stage, the minimum essential information known through environmental factors or pre-exchange processes before executing the proposed protocol is defined. In the goal stage, the security goals are defined to require in the target environment of the proposed protocol. Finally, in the derivation stage, a series of processes are represented to derive the security properties defined in the goal stage through BAN logic’s rules presented in Table 4 from the results of the idealization and assumption stages. The results of verifying the security of the proposed security protocol through BAN logic in this paper are as follows.

#### 4.1.1. Idealization

As a first step, the proposed security protocol in this paper is modeled in an idealization form that removes unnecessary elements other than the message protected by encryption and message authentication codes. The idealization form of the proposed security protocol is presented below:

(1) $$\begin{array}{cc} \mathrm{CONT}\to \mathrm{CGM}\;:\; & {\langle Sid,g^{x},n_{1},n_{2},\mathrm{CONT}\overset{PWD_{CGM}}{↔}\mathrm{CGM}\rangle }_{PWD_{CGM}} \end{array}$$

(2) $$\begin{array}{cc} \mathrm{CGM}\to \mathrm{CONT}\;:\; & {\langle Sid,g^{y},n_{2},n_{3},\mathrm{CONT}\overset{PWD_{CGM}}{↔}\mathrm{CGM}|\mathrm{CONT}\overset{SK_{CGM}}{↔}\mathrm{CGM}\rangle }_{PWD_{CGM}} \end{array}$$

(3) $$\begin{array}{cc} \mathrm{CONT}\to \mathrm{IP}\;: & {\langle Sid,g^{x},n_{4},n_{5},\mathrm{CONT}\overset{PWD_{IP}}{↔}\mathrm{IP}\rangle }_{PWD_{IP}} \end{array}$$

(4) $$\begin{array}{cc} \mathrm{IP}\to \mathrm{CONT}\;: & {\langle Sid,g^{z},n_{5},n_{6},\mathrm{CONT}\overset{PWD_{IP}}{↔}\mathrm{IP},\mathrm{CONT}\overset{SK_{IP}}{↔}\mathrm{IP}\rangle }_{SK_{IP}} \end{array}$$

(5) $$\begin{array}{cc} \mathrm{CONT}\to \mathrm{CGM}\;: & {\langle Sid,g^{z},n_{3},n_{6},\mathrm{CONT}\overset{SK_{CGM}}{↔}\mathrm{CGM}\rangle }_{SK_{CGM}} \end{array}$$

(6) $$\begin{array}{cc} \mathrm{CONT}\to \mathrm{IP}\;: & {\langle Sid,g^{y},n_{3},n_{6},\mathrm{CONT}\overset{SK_{IP}}{↔}\mathrm{IP}\rangle }_{SK_{IP}} \end{array}$$

(7) $$\begin{array}{cc} \mathrm{IP}\to \mathrm{CGM}\;: & {\langle Sid,Alert,\mathrm{CGM}\overset{SK_{EMG}}{↔}\mathrm{IP}\rangle }_{SK_{EMG}} \end{array}$$

(8) $$\begin{array}{cc} \mathrm{CGM}\to \mathrm{IP}\;: & {\langle Sid,BGL,\mathrm{CGM}\overset{SK_{EMG}}{↔}\mathrm{IP}\rangle }_{SK_{EMG}} \end{array}$$

#### 4.1.2. Assumptions

In the Assumption step, assumptions necessary for the operation of the proposed protocol are formulated. Assumptions should contain only the minimum necessary and realistic information. The assumptions used in this section define security keys and freshness for CGM, CONT, and IP as follows:

(9) $$\begin{array}{cc} \; & \mathrm{CGM}|\equiv \mathrm{CONT}\overset{PWD_{CGM}}{↔}\mathrm{CGM} \end{array}$$

(10) $$\begin{array}{cc} \; & \mathrm{CGM}|\equiv \#(n_{1}) \end{array}$$

(11) $$\begin{array}{cc} \; & \mathrm{CGM}|\equiv \overset{g^{y}}{⟼}\mathrm{CGM} \end{array}$$

(12) $$\begin{array}{cc} \; & \mathrm{CONT}|\equiv \mathrm{CONT}\overset{PWD_{CGM}}{↔}\mathrm{CGM} \end{array}$$

(13) $$\begin{array}{cc} \; & \mathrm{CONT}|\equiv \#(n_{2}) \end{array}$$

(14) $$\begin{array}{cc} \; & \mathrm{CONT}|\equiv \overset{g^{x}}{⟼}\mathrm{CONT} \end{array}$$

(15) $$\begin{array}{cc} \; & \mathrm{IP}|\equiv \mathrm{CONT}\overset{PWD_{IP}}{↔}\mathrm{IP} \end{array}$$

(16) $$\begin{array}{cc} \; & \mathrm{IP}|\equiv \#(n_{4}) \end{array}$$

(17) $$\begin{array}{cc} \; & \mathrm{IP}|\equiv \overset{g^{z}}{⟼}\mathrm{IP} \end{array}$$

(18) $$\begin{array}{cc} \; & \mathrm{CONT}|\equiv \mathrm{CONT}\overset{PWD_{IP}}{↔}\mathrm{IP} \end{array}$$

(19) $$\begin{array}{cc} \; & \mathrm{CONT}|\equiv \#(n_{5}) \end{array}$$

(20) $$\begin{array}{cc} \; & \mathrm{CGM}|\equiv \#(n_{3}) \end{array}$$

(21) $$\begin{array}{cc} \; & \mathrm{IP}|\equiv \#(n_{6}) \end{array}$$

#### 4.1.3. Goals

Security protocols must meet security requirements to ensure safe communication for users in the environment in which they are applied. The security protocol proposed in this paper aims to protect the user’s sensitive medical information from malicious users in the APS environment. Therefore, we verify the security of the user authentication and session key exchange process presented in the proposed security protocol. The following defines the security requirements necessary to provide secure communication in the APS environment, such as user authentication and key reliability.

(22) $$\begin{array}{cc} \; & \mathrm{CGM}|\equiv \mathrm{CONT}|\equiv \mathrm{CONT}\overset{PWD_{CGM}}{↔}\mathrm{CGM} \end{array}$$

(23) $$\begin{array}{cc} \; & \mathrm{CGM}|\equiv \mathrm{CONT}\overset{g^{xy}}{↔}\mathrm{CGM} \end{array}$$

(24) $$\begin{array}{cc} \; & \mathrm{CGM}|\equiv \mathrm{CONT}\overset{SK_{CGM}}{↔}\mathrm{CGM} \end{array}$$

(25) $$\begin{array}{cc} \; & \mathrm{CONT}|\equiv \mathrm{CGM}|\equiv \mathrm{CONT}\overset{PWD_{CGM}}{↔}\mathrm{CGM} \end{array}$$

(26) $$\begin{array}{cc} \; & \mathrm{CONT}|\equiv \mathrm{CGM}|\equiv \mathrm{CONT}\overset{SK_{CGM}}{↔}\mathrm{CGM} \end{array}$$

(27) $$\begin{array}{cc} \; & \mathrm{CONT}|\equiv \mathrm{CONT}\overset{g^{xy}}{↔}\mathrm{CGM} \end{array}$$

(28) $$\begin{array}{cc} \; & \mathrm{CONT}|\equiv \mathrm{CONT}\overset{SK_{CGM}}{↔}\mathrm{CGM} \end{array}$$

(29) $$\begin{array}{cc} \; & \mathrm{IP}|\equiv \mathrm{CONT}|\equiv \mathrm{CONT}\overset{PWD_{IP}}{↔}\mathrm{IP} \end{array}$$

(30) $$\begin{array}{cc} \; & \mathrm{IP}|\equiv \mathrm{CONT}\overset{g^{xz}}{↔}\mathrm{IP} \end{array}$$

(31) $$\begin{array}{cc} \; & \mathrm{IP}|\equiv \mathrm{CONT}\overset{SK_{IP}}{↔}\mathrm{IP} \end{array}$$

(32) $$\begin{array}{cc} \; & \mathrm{CONT}|\equiv \mathrm{IP}|\equiv \mathrm{CONT}\overset{PWD_{IP}}{↔}\mathrm{IP} \end{array}$$

(33) $$\begin{array}{cc} \; & \mathrm{CONT}|\equiv \mathrm{IP}|\equiv \mathrm{CONT}\overset{SK_{IP}}{↔}\mathrm{IP} \end{array}$$

(34) $$\begin{array}{cc} \; & \mathrm{CONT}|\equiv \mathrm{CONT}\overset{g^{xz}}{↔}\mathrm{IP} \end{array}$$

(35) $$\begin{array}{cc} \; & \mathrm{CONT}|\equiv \mathrm{CONT}\overset{SK_{IP}}{↔}\mathrm{IP} \end{array}$$

(36) $$\begin{array}{cc} \; & \mathrm{CGM}|\equiv \mathrm{CONT}|\equiv \mathrm{CONT}\overset{SK_{CGM}}{↔}\mathrm{CGM} \end{array}$$

(37) $$\begin{array}{cc} \; & \mathrm{CGM}|\equiv \mathrm{CGM}\overset{g^{yz}}{↔}\mathrm{IP} \end{array}$$

(38) $$\begin{array}{cc} \; & \mathrm{CGM}|\equiv \mathrm{CGM}\overset{SK_{EMG}}{↔}\mathrm{IP} \end{array}$$

(39) $$\begin{array}{cc} \; & \mathrm{IP}|\equiv \mathrm{CONT}|\equiv \mathrm{CONT}\overset{SK_{IP}}{↔}\mathrm{IP} \end{array}$$

(40) $$\begin{array}{cc} \; & \mathrm{IP}|\equiv \mathrm{CGM}\overset{g^{yz}}{↔}\mathrm{IP} \end{array}$$

(41) $$\begin{array}{cc} \; & \mathrm{IP}|\equiv \mathrm{CGM}\overset{SK_{EMG}}{↔}\mathrm{IP} \end{array}$$

(42) $$\begin{array}{cc} \; & \mathrm{IP}|\equiv \mathrm{CGM}|\equiv \mathrm{CGM}\overset{SK_{EMG}}{↔}\mathrm{IP} \end{array}$$

(43) $$\begin{array}{cc} \; & \mathrm{CGM}|\equiv \mathrm{IP}|\equiv \mathrm{CGM}\overset{SK_{EMG}}{↔}\mathrm{IP} \end{array}$$

#### 4.1.4. Derivation

Finally, the proposed security protocol is analyzed by applying the idealization form (1–8) and assumptions (9–22) to the rules of BAN logic (Table 4). The results derived from each idealization form must be appropriately matched with the Goals (i.e., security requirements) required by the target environment. The detailed derivation process for verifying the security of the protocol is as follows.

From Idealization (1):

(44) $$\begin{array}{cccc} & \mathrm{CGM}◃{\langle Sid,\overset{g^{x}}{⟼}\mathrm{CONT},n_{1},n_{2},\mathrm{CONT}\overset{PWD_{CGM}}{↔}\mathrm{CGM}\rangle }_{PWD_{CGM}}\;\mathrm{by}\;\left(1\right) & & \end{array}$$

(45) $$\begin{array}{cccc} & \mathrm{CGM}|\equiv \mathrm{CONT}|∼\left(Sid,\overset{g^{x}}{⟼}\mathrm{CONT},n_{1},n_{2},\mathrm{CONT}\overset{PWD_{CGM}}{↔}\mathrm{CGM}\right) & \\ & \;\;\;\;\;\;\;\;\;\;\;\;\mathrm{by}\;(44),\;(9),\;\mathrm{MM} & & \end{array}$$

(46) $$\begin{array}{ccc} & \mathrm{CGM}|\equiv \mathrm{CONT}|\equiv \left(Sid,\overset{g^{x}}{⟼}\mathrm{CONT},n_{1},n_{2},\mathrm{CONT}\overset{PWD_{CGM}}{↔}\mathrm{CGM}\right) & \\ & \;\;\;\;\;\;\;\;\;\;\;\;\mathrm{by}\;(45),\;(10),\;\mathrm{FR},\;\mathrm{NV} \end{array}$$

(47) $$\begin{array}{cccc} & \mathrm{CGM}|\equiv \mathrm{CONT}|\equiv \mathrm{CONT}\overset{PWD_{CGM}}{↔}\mathrm{CGM}\;\mathrm{by}\;(46),\;\mathrm{BC} & & \end{array}$$

(48) $$\begin{array}{cccc} & \mathrm{CGM}|\equiv \mathrm{CONT}|∼\overset{g^{x}}{⟼}\mathrm{CONT}\;\mathrm{by}\;(45),\;\mathrm{BC} & & \end{array}$$

(49) $$\begin{array}{cccc} & \mathrm{CGM}|\equiv \mathrm{CONT}\overset{g^{xy}}{↔}\mathrm{CGM}\;\mathrm{by}\;(48),\;(11),\;\mathrm{DH} & & \end{array}$$

(50) $$\begin{array}{ccc} & \mathrm{CGM}|\equiv \mathrm{CONT}\overset{SK_{CGM}}{↔}\mathrm{CGM}\;\mathrm{by}\;(49),\;(46),\;\mathrm{BC} & \end{array}$$

From Idealization (2):

(51) $$\begin{array}{ccc} & \mathrm{CONT}◃{\langle Sid,\overset{g^{y}}{⟼}\mathrm{CGM},n_{2},n_{3},\mathrm{CONT}\overset{PWD_{CGM}}{↔}\mathrm{CGM},\mathrm{CONT}\overset{SK_{CGM}}{↔}\mathrm{CGM}\rangle }_{PWD_{CGM}} & \\ & \;\;\;\;\;\;\;\;\;\;\;\;\mathrm{by}\;\left(2\right) \end{array}$$

(52) $$\begin{array}{ccc} & \mathrm{CONT}|\equiv \mathrm{CGM}|∼\left(Sidm\overset{g^{y}}{⟼}\mathrm{CGM},n_{2},n_{3},\mathrm{CONT}\overset{PWD_{CGM}}{↔}\mathrm{CGM}|\mathrm{CONT}\overset{SK_{CGM}}{↔}\mathrm{CGM}\right) & \\ & \;\;\;\;\;\;\;\;\;\;\;\;\mathrm{by}\;(51),\;(12),\;\mathrm{MM} \end{array}$$

(53) $$\begin{array}{ccc} & \mathrm{CONT}|\equiv \mathrm{CGM}|\equiv \left(Sid,\overset{g^{y}}{⟼}\mathrm{CGM},n_{2},n_{3},\mathrm{CONT}\overset{PWD_{CGM}}{↔}\mathrm{CGM},\mathrm{CONT}\overset{SK_{CGM}}{↔}\mathrm{CGM}\right) & \\ & \;\;\;\;\;\;\;\;\;\;\;\;\mathrm{by}\;(52),\;(13),\;\mathrm{FR},\;\mathrm{NV} \end{array}$$

(54) $$\begin{array}{ccc} & \mathrm{CONT}|\equiv \mathrm{CGM}|\equiv \mathrm{CONT}\overset{PWD_{CGM}}{↔}\mathrm{CGM}\;\mathrm{by}\;(53),\;\mathrm{BC} & \end{array}$$

(55) $$\begin{array}{ccc} & \mathrm{CONT}|\equiv \mathrm{CGM}|\equiv \mathrm{CONT}\overset{SK_{CGM}}{↔}\mathrm{CGM}\;\mathrm{by}\;(53),\;\mathrm{BC} & \end{array}$$

(56) $$\begin{array}{ccc} & \mathrm{CONT}|\equiv \mathrm{CGM}|∼\overset{g^{y}}{⟼}\mathrm{CGM}\;\mathrm{by}\;(52),\;\mathrm{BC} & \end{array}$$

(57) $$\begin{array}{ccc} & \mathrm{CONT}|\equiv \mathrm{CONT}\overset{g^{xy}}{↔}\mathrm{CGM}\;\mathrm{by}\;(56),\;(14),\;\mathrm{DH} & \end{array}$$

(58) $$\begin{array}{cc} & \mathrm{CONT}|\equiv \mathrm{CONT}\overset{SK_{CGM}}{↔}\mathrm{CGM}\;\mathrm{by}\;(57),\;(18),\;\mathrm{BC} \end{array}$$

From Idealization (3):

(59) $$\begin{array}{ccc} & \mathrm{IP}◃{\langle Sid,g^{x},n_{4},n_{5},PWD_{IP}\rangle }_{PWD_{IP}}\;\mathrm{by}\;\left(3\right) & \end{array}$$

(60) $$\begin{array}{ccc} & \mathrm{IP}|\equiv \mathrm{CONT}|∼\left(Sid,g^{x},n_{4},n_{5},PWD_{IP}\right)\;\mathrm{by}\;(59),\;(15),\;\mathrm{MM} & \end{array}$$

(61) $$\begin{array}{ccc} & \mathrm{IP}|\equiv \mathrm{CONT}|\equiv \left(Sid,g^{x},n_{4},n_{5},PWD_{IP}\right)\;\mathrm{by}\;(60),\;(16),\;\mathrm{FR},\;\mathrm{NV} & \end{array}$$

(62) $$\begin{array}{ccc} & \mathrm{IP}|\equiv \mathrm{CONT}|\equiv \mathrm{CONT}\overset{PWD_{IP}}{↔}\mathrm{IP}\;\mathrm{by}\;(61),\;\mathrm{BC} & \end{array}$$

(63) $$\begin{array}{c} \mathrm{IP}|\equiv \mathrm{CONT}|∼\overset{g^{x}}{⟼}\mathrm{CONT}\;\mathrm{by}\;(60),\;\mathrm{BC} \end{array}$$

(64) $$\begin{array}{c} \mathrm{IP}|\equiv \mathrm{CONT}\overset{g^{xz}}{↔}\mathrm{IP}\;\mathrm{by}\;(63),\;(A9),\;\mathrm{DH} \end{array}$$

(65) $$\begin{array}{c} \mathrm{IP}|\equiv \mathrm{CONT}\overset{SK_{IP}}{↔}\mathrm{IP}\;\mathrm{by}\;(64),\;(61),\;\mathrm{BC} \end{array}$$

From Idealization (4):

(66) $$\begin{array}{c} \mathrm{CONT}◃{\langle Sid,\overset{g^{z}}{⟼}\mathrm{IP},n_{5},n_{6},\mathrm{CONT}\overset{PWD_{IP}}{↔}\mathrm{IP},\mathrm{CONT}\overset{SK_{IP}}{↔}\mathrm{IP}\rangle }_{PWD_{IP}}\;\mathrm{by}\;\left(4\right) \end{array}$$

(67) $$\begin{array}{c} \mathrm{CONT}|\equiv \mathrm{IP}|∼\left(Sid,\overset{g^{z}}{⟼}\mathrm{IP},n_{5},n_{6},\mathrm{CONT}\overset{PWD_{IP}}{↔}\mathrm{IP},\mathrm{CONT}\overset{SK_{IP}}{↔}\mathrm{IP}\right)\\ \;\;\;\;\;\;\;\;\;\;\;\;\mathrm{by}\;(66),\;(18),\;\mathrm{MM} \end{array}$$

(68) $$\begin{array}{cc} \mathrm{CONT}|\equiv \mathrm{IP}|\equiv \left(Sid,\overset{g^{z}}{⟼}\mathrm{IP},n_{5},n_{6},\mathrm{CONT}\overset{PWD_{IP}}{↔}\mathrm{IP},\mathrm{CONT}\overset{SK_{IP}}{↔}\mathrm{IP}\right)\\ \;\;\;\;\;\;\;\;\;\;\;\;\mathrm{by}\;(67),\;(19),\;\mathrm{FR},\;\mathrm{NV} & \end{array}$$

(69) $$\begin{array}{c} \mathrm{CONT}|\equiv \mathrm{IP}|\equiv \mathrm{CONT}\overset{PWD_{IP}}{↔}\mathrm{IP}\;\mathrm{by}\;(68),\;\mathrm{BC} \end{array}$$

(70) $$\begin{array}{c} \mathrm{CONT}|\equiv \mathrm{IP}|\equiv \mathrm{CONT}\overset{SK_{IP}}{↔}\mathrm{IP}\;\mathrm{by}\;(68),\;\mathrm{BC} \end{array}$$

(71) $$\begin{array}{c} \mathrm{CONT}|\equiv \mathrm{IP}|∼\overset{g^{z}}{⟼}\mathrm{IP}\;\mathrm{by}\;(67),\;\mathrm{BC} \end{array}$$

(72) $$\begin{array}{c} \mathrm{CONT}|\equiv \mathrm{CONT}\overset{g^{xz}}{↔}\mathrm{IP}\;\mathrm{by}\;(71),\;(14),\;\mathrm{DH} \end{array}$$

(73) $$\begin{array}{c} \mathrm{CONT}|\equiv \mathrm{CONT}\overset{SK_{IP}}{↔}\mathrm{IP}\;\mathrm{by}\;(72),\;(68),\;\mathrm{BC} \end{array}$$

From Idealization (5):

(74) $$\begin{array}{cc} & \mathrm{CGM}◃{\langle Sid,\overset{g^{z}}{⟼}\mathrm{IP},n_{3},n_{6},\mathrm{CONT}\overset{SK_{CGM}}{↔}\mathrm{CGM}\rangle }_{SK_{CGM}}\;\mathrm{by}\;\left(5\right) \end{array}$$

(75) $$\begin{array}{cc} \mathrm{CGM}|\equiv \mathrm{CONT}|∼\left(Sid,\overset{g^{z}}{⟼}\mathrm{IP},n_{3},n_{6},\mathrm{CONT}\overset{SK_{CGM}}{↔}\mathrm{CGM}\right)\;\mathrm{by}\;(74),\;(50),\;\mathrm{MM} & \end{array}$$

(76) $$\begin{array}{c} \mathrm{CGM}|\equiv \mathrm{CONT}|\equiv \left(Sid,\overset{g^{z}}{⟼}\mathrm{IP},n_{3},n_{6},\mathrm{CONT}\overset{SK_{CGM}}{↔}\mathrm{CGM}\right)\;\mathrm{by}\;(75),\;(20),\;\mathrm{FR},\;\mathrm{NV} \end{array}$$

(77) $$\begin{array}{c} \mathrm{CGM}|\equiv \mathrm{CONT}|\equiv \mathrm{CONT}\overset{SK_{CGM}}{↔}\mathrm{CGM}\;\mathrm{by}\;(76),\;\mathrm{BC} \end{array}$$

(78) $$\begin{array}{c} \mathrm{CGM}|\equiv \mathrm{CONT}|∼\overset{g^{z}}{⟼}\mathrm{IP}\;\mathrm{by}\;(75),\;\mathrm{BC} \end{array}$$

(79) $$\begin{array}{c} \mathrm{CGM}|\equiv \mathrm{CGM}\overset{g^{yz}}{↔}\mathrm{IP}\;\mathrm{by}\;(78),\;(11),\;\mathrm{DH} \end{array}$$

(80) $$\begin{array}{c} \mathrm{CGM}|\equiv \mathrm{CGM}\overset{SK_{EMG}}{↔}\mathrm{IP}\;\mathrm{by}\;(79),\;(76),\;\mathrm{BC} \end{array}$$

(81) $$\begin{array}{c} \mathrm{CGM}|\equiv \#(\mathrm{CGM}\overset{SK_{EMG}}{↔}\mathrm{IP})\;\mathrm{by}\;(79),\;(76),\;\mathrm{BC} \end{array}$$

From Idealization (6):

(82) $$\begin{array}{ccc} & \mathrm{IP}◃{\langle Sid,\overset{g^{y}}{⟼}\mathrm{CGM},n_{3},n_{6},\mathrm{CONT}\overset{SK_{IP}}{↔}\mathrm{IP}\rangle }_{SK_{IP}}\;\mathrm{by}\;\left(6\right) & \end{array}$$

(83) $$\begin{array}{c} \mathrm{IP}|\equiv \mathrm{CONT}|∼\left(Sid,\overset{g^{y}}{⟼}\mathrm{CGM},n_{3},n_{6},\mathrm{CONT}\overset{SK_{IP}}{↔}\mathrm{IP}\right)\;\mathrm{by}\;(82),\;(65),\;\mathrm{MM} \end{array}$$

(84) $$\begin{array}{c} \mathrm{IP}|\equiv \mathrm{CONT}|\equiv \left(Sid,\overset{g^{y}}{⟼}\mathrm{CGM},n_{3},n_{6},\mathrm{CONT}\overset{SK_{IP}}{↔}\mathrm{IP}\right)\;\mathrm{by}\;(83),\;(21),\;\mathrm{FR},\;\mathrm{NV} \end{array}$$

(85) $$\begin{array}{c} \mathrm{IP}|\equiv \mathrm{CONT}|\equiv \mathrm{CONT}\overset{SK_{IP}}{↔}\mathrm{IP}\;\mathrm{by}\;(84),\;\mathrm{BC} \end{array}$$

(86) $$\begin{array}{c} \mathrm{IP}|\equiv \mathrm{CONT}|∼\overset{g^{y}}{⟼}\mathrm{CGM}\;\mathrm{by}\;(83),\;\mathrm{BC} \end{array}$$

(87) $$\begin{array}{c} \mathrm{IP}|\equiv \mathrm{CGM}\overset{g^{yz}}{↔}\mathrm{IP}\;\mathrm{by}\;(86),\;(17),\;\mathrm{DH} \end{array}$$

(88) $$\begin{array}{c} \mathrm{IP}|\equiv \mathrm{CGM}\overset{SK_{EMG}}{↔}\mathrm{IP}\;\mathrm{by}\;(87),\;(83),\;\mathrm{BC} \end{array}$$

(89) $$\begin{array}{c} \mathrm{IP}|\equiv \#(\mathrm{CGM}\overset{SK_{EMG}}{↔}\mathrm{IP})\;\mathrm{by}\;(87),\;(83),\;\mathrm{BC} \end{array}$$

From Idealization (7):

(90) $$\begin{array}{cc} \mathrm{CGM}◃{\langle Sid,Alert,\mathrm{CGM}\overset{SK_{EMG}}{↔}\mathrm{IP}\rangle }_{SK_{EMG}}\;\mathrm{by}\;\left(7\right) & \end{array}$$

(91) $$\begin{array}{c} \mathrm{CGM}|\equiv \mathrm{IP}|∼\left(Sid,Alert,\mathrm{CGM}\overset{SK_{EMG}}{↔}\mathrm{IP}\right)\;\mathrm{by}\;(90),\;(80),\;\mathrm{MM} \end{array}$$

(92) $$\begin{array}{c} \mathrm{CGM}|\equiv \mathrm{IP}|\equiv \left(Sid,Alert,\mathrm{CGM}\overset{SK_{EMG}}{↔}\mathrm{IP}\right)\;\mathrm{by}\;(91),\;(81),\;\mathrm{FR},\;\mathrm{NV} \end{array}$$

(93) $$\begin{array}{c} \mathrm{CGM}|\equiv \mathrm{IP}|\equiv \mathrm{CGM}\overset{SK_{EMG}}{↔}\mathrm{IP}\;\mathrm{by}\;(92),\;\mathrm{BC} \end{array}$$

From Idealization (8):

(94) $$\begin{array}{ccc} & \mathrm{IP}◃{\langle Sid,BGL,\mathrm{CGM}\overset{SK_{EMG}}{↔}\mathrm{IP}\rangle }_{SK_{EMG}}\;\mathrm{by}\;\left(8\right) & \end{array}$$

(95) $$\begin{array}{c} \mathrm{IP}|\equiv \mathrm{CGM}|∼\left(Sid,BGL,\mathrm{CGM}\overset{SK_{EMG}}{↔}\mathrm{IP}\right)\;\mathrm{by}\;(94),\;(88),\;\mathrm{MM} \end{array}$$

(96) $$\begin{array}{c} \mathrm{IP}|\equiv \mathrm{CGM}|\equiv \left(Sid,BGL,\mathrm{CGM}\overset{SK_{EMG}}{↔}\mathrm{IP}\right)\;\mathrm{by}\;(95),\;(89),\;\mathrm{FR},\;\mathrm{NV} \end{array}$$

(97) $$\begin{array}{c} \mathrm{IP}|\equiv \mathrm{CGM}|\equiv \mathrm{CGM}\overset{SK_{EMG}}{↔}\mathrm{IP}\;\mathrm{by}\;(96),\;\mathrm{BC} \end{array}$$

According to the derivation above, the proposed security protocol can achieve all the security goals stated in the goals. This means that the proposed protocol can securely protect users’ medical information from malicious attackers. Table 5 compares the security requirements, the security goals presented in the goals, and the stages of derivation at which the security goals are achieved. Furthermore, we show that the security requirements and goals are satisfied by using Theorem 1 and following Lemmas.

**Theorem** **1.** *The proposed security protocol is secure against malicious attacks.*

**Proof** **of** **Theorem** **1.** Through Lemma 1 to Lemma 6, the defined goals are satisfied that the proposed security protocol is secure against malicious attacks. □

**Lemma** **1.** *The proposed security protocol can provide mutual authentication in three communication segments, CONT and CGM, CONT and IP, and IP and CGM.*

**Proof** **of** **Lemma** **1.** On the one hand, it can be demonstrated that the security protocol supports mutual authentication by verifying the trust between each other through a pre-shared key. In our proposed protocol, CGM and IP each use a pre-shared key with CONT. Mutual authentication between CGM and CONT can be verified through derivations (47) and (54). Similarly, mutual authentication between IP and CONT can be verified through derivations (62) and (69). On the other hand, there is no pre-shared key between CGM and IP. However, mutual authentication can be demonstrated based on the trust of the emergency session key $SK_{EMG}$, which was exchanged during the Registration phase. □

**Lemma** **2.** *The session keys $SK_{CGM}$ and $SK_{IP}$ are securely exchanged between CGM and CONT, and between IP and CONT, respectively.*

**Proof** **of** **Lemma** **2.** CGM and CONT have direct beliefs in the session key $SK_{CGM}$ through the derivations (50) and (58). Furthermore, they can trust that each other has belief in the session key $SK_{CGM}$ through the derivation (55) and (69). Similarly, IP and CONT can verify their direct beliefs in the session key $SK_{IP}$ through the derivations (65) and (73). Their indirect beliefs in the session key can be confirmed through the derivations (70) and (85). □

**Lemma** **3.** *The proposed security protocol can provide the perfect forward secrecy in the session keys $SK_{CGM}$ and $SK_{IP}$.*

**Proof** **of** **Lemma** **3.** From (49) and (57), CGM and CONT derive the session key $SK_{CGM}$ through ECDHE key exchange. ECDHE key exchange utilizes the ephemeral key, and, thus, the private key is deleted immediately after the key exchange. This prevents the restoration of future keys and eliminates the relationship between keys, allowing for perfect forward secrecy. The session key $SK_{IP}$ also satisfies perfect forward secrecy through ECDHE key exchange between IP and CONT from (64) and (72). □

**Lemma** **4.** *The proposed security protocol can provide a secure channel between CGM and IP in emergency situations.*

**Proof** **of** **Lemma** **4.** The proposed security protocol in this paper provides not only the Registration phase and the Communication phase but also the Emergency phase. During the Emergency phase, CGM and IP can communicate securely using the emergency session key exchanged in the Registration phase. In the above derivations, CGM and IP can verify both direct beliefs (80), (88) and indirect beliefs (93), (97) for the emergency session key $SK_{EMG}$. □

**Lemma** **5.** *The proposed security protocol can provide the perfect forward secrecy in the emergency session key ($SK_{EMG}$).*

**Proof** **of** **Lemma** **5.** According to (79) and (87), the emergency session key $SK_{EMG}$ is generated with the ECDHE session key. As same as Lemma 3, the emergency session key also satisfies the perfect forward secrecy. □

**Lemma** **6.** *The proposed security protocol can provide confidentiality and integrity.*

**Proof** **of** **Lemma** **6.** The confidentiality of the protocol depends on the secure exchange of keys and the safety of the keys themselves. The security protocol proposed in this paper has demonstrated secure key exchange for the session keys $SK_{CGM}$, $SK_{IP}$, and $SK_{EMG}$ in Lemma 2 and Lemma 4. In addition, Lemma 3 and Lemma 5 have confirmed that perfect forward secrecy is provided for each key. Therefore, the session keys exchanged in the proposed security protocol are secure and can provide confidentiality. As confirmed by Lemma 1, CGM, IP, and CONT all have both direct and indirect trust in the pre-shared keys, allowing them to believe that messages have not been altered during transmission. □

As a result, the safety of the proposed security protocol has been proven through the Theorem and Lemmas presented in this paper, and it can be considered secure from malicious attacks.

### 4.2. AVISPA

In the previous section, we proved the security of the proposed security protocol using BAN logic. BAN logic is a useful tool for representing and analyzing security protocols based on modal logic. However, previous research has pointed out the limitations of BAN logic, such as inaccurate message representation and the absence of inference rules for hash functions, in clearly transforming security protocols into an idealization form. While acknowledging that BAN logic is a useful formal verification tool, this research recommends using automated formal verification tools in conjunction with BAN logic [21,22]. Therefore, in this paper, we verify the security of the proposed security protocol using AVISPA [20], an automated formal verification tool, in conjunction with BAN Logic.

**Figure 3:**
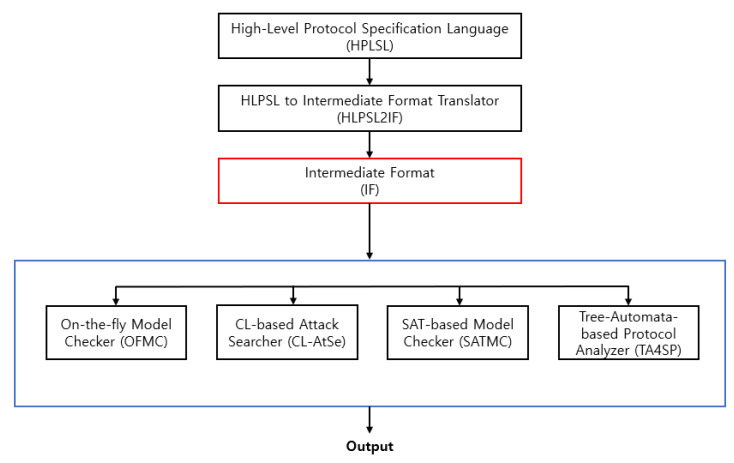
Architecture of AVISPA.

AVISPA provides a toolset that includes a high-level protocol specification language (HLPSL) [23] to describe security protocols and to specify intended security properties. HLPSL2IF converts HLPSL specifications into an intermediate format (IF), which is a low-level language directly read by the backend of AVISPA tools. The IF specification of the protocol is then input to the backend of the AVISPA tool to analyze the specified security goals. Figure 3 illustrates this process.

**Figure 4:**
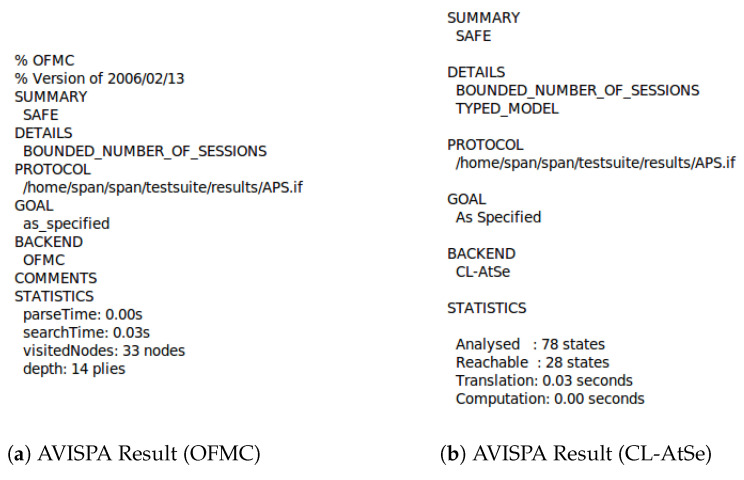
AVISPA results of the proposed security protocol.

**Figure 5:**
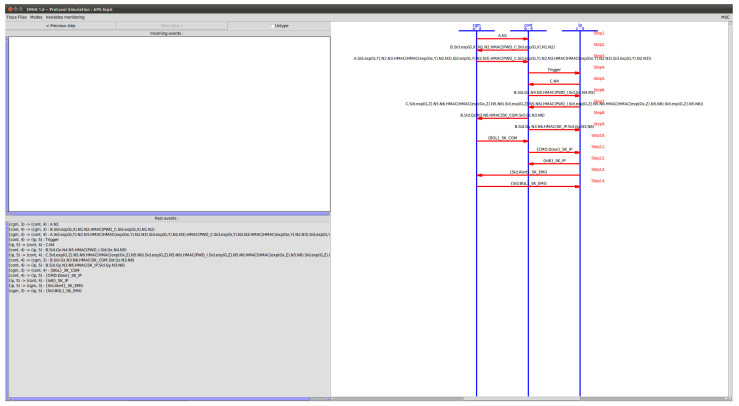
AVISPA protocol simulation.

The HLPSL specification consists of three modules, basic roles used in the roles, session, and environment. The basic roles represent the specifications of each protocol participant modeled with known initial information as parameters. Then, these roles are invoked to specify how the resulting participants interact by linking various basic roles to the configured roles. The transition part of the HLPSL specification includes a set of transitions between different roles. Each transition symbolizes message acceptance and response message transmission. Figure 4 a,b, and Figure 5 show the AVISPA verification results and protocol simulation results for the protocol. Since the results obtained from both options of AVISPA, the proposed security protocol has been analyzed as ’SAFE’, which can be seen as secure against known attacks.

### 4.3. Discussion

In this section, we present the comparison evaluation results based on two aspects, security analysis and overhead analysis. For the comparison, we have adopted not only IMD security protocols (Wu et al. [13], Chi et al. [14], HAT [15]) presented in Related Works but also widely adopted protocols in communication devices, such as Bluetooth [16] and TLS 1.3 [18]. In the security analysis, we compare the support for seven security requirements. In the overhead analysis, we implement the system as described in Section 5 and analyze the Computational Overhead and Communication Overhead based on the implementation.

#### 4.3.1. Security Analysis

The proposed security protocol is compared to existing protocols in terms of the seven security requirements (Mutual Authentication, Confidentiality, Integrity, Secure Key Exchange, Perfect Forward Secrecy, Privacy, and Emergency Phase) presented in Section 3. The security requirements that each security protocol satisfies are shown in Table 6.

According to Table 6, all protocols can support Mutual Authentication (AUTH), Confidentiality (CONF), Integrity (INT), and Secure Key Exchange (SKE). Perfect Forward Secrecy (PFS) was satisfied by HAT, TLS 1.3, and the proposed protocol and Privacy (PRV) can be provided by TLS 1.3 and the proposed protocol. It can be observed that the Emergency phase (EMEG), which was presented as an important security requirement of APS in this paper, is only satisfied by the proposed protocol. Finally, all protocols except for Bluetooth LE can provide countermeasures against Replay Attacks (RPA) and Man-in-The-Middle Attacks (MiTM). In summary, only the proposed protocol can satisfy all seven security requirements presented in this paper.

#### 4.3.2. Overhead Analysis

To the best of our knowledge, there is limited meaningful research on security protocols focused on APS, and this paper aims to pioneer this area by designing and verifying a security protocol. To demonstrate the feasibility of the proposed security protocol, we conduct a comparative analysis of computational (Table 7) and communication (Table 8) overheads with existing studies designed for IMD, a field similar to APS.

Table 7 and Table 8 compare our proposed protocol with existing IMD security protocols, as well as Bluetooth LE and TLS 1.3, in terms of computational and communication overheads. Compared with [13,14,16,18], our proposed protocol is more efficient due to the exclusion of asymmetric key operations based on the characteristics of APS, which is based on low-performance devices. On the other hand, our proposed protocol may have higher computational costs than [15] due to the additional computations required for exchanging the session keys for Emergency phase only supported by our proposed protocol.

## 5. Experiment

To prove the feasibility of the proposed protocol, we have set up the environment for the APS and implemented the protocol. We used the Arduino Nano 33 kit for the Insulin pump, nRF52840 development kit (DK) for CGM, and Galaxy S20 series with Android 13 for the controller. Detailed specifications of the implementation environment are shown in Table 9.

**Figure 6:**
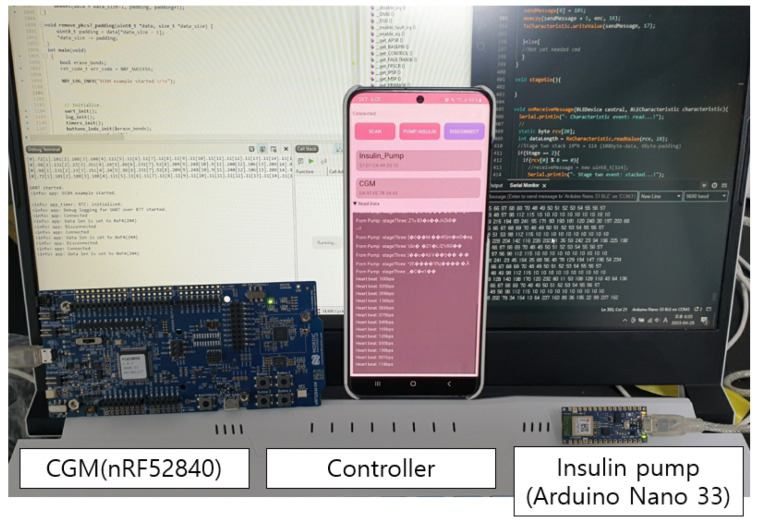
Experimental emulation for the APS.

Figure 6 shows the implemented emulation for the APS. Note that this emulation is based on a real product on a market to enhance the credibility of the test. Though we have proposed our protocol, hoping to replace the security protocol of Bluetooth, we have implemented the protocol on the application layer, which is on top of the Bluetooth layer, for fast testing purposes.

After successfully implementing our proposed protocol, we conducted a test to compare the computation overhead and energy consumption with the widely used BLE mode in the IoT industry. This comparison is important for assessing feasibility. To ensure accuracy, we needed to standardize the test scenario. Our proposed protocol establishes secure channels among IP, CONT, and CGM at once. However, BLE only supports one-to-one communication. Therefore, we assumed three separate BLE connections in order to compare with the proposed protocol in the same APS environment. In other words, each device should establish two BLE connections. The following Table 10 presents the computation overhead in each environment based on a unified scenario.

**Figure 7:**
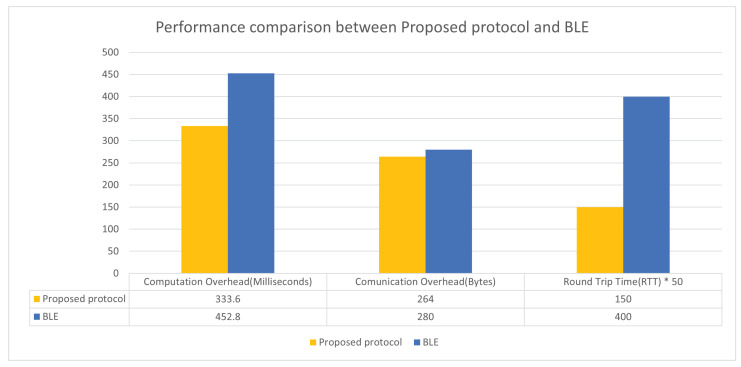
Comparison of two protocols on Arduino Nano 33.

The experimental comparison shows significantly different results depending on the processor. IoT devices took more than 400 times longer to handle cryptographical computation. This means that feasibility is much more dependent on how much it is suitable for IoT devices, not the controller. Figure 7 shows a more detailed comparison of our protocol and BLE.

In this section, we present a comparative analysis between our proposed protocol and Bluetooth Low Energy (BLE) regarding the computational overhead incurred during the Registration phase. The APS environment, as outlined in this paper, consists of a closed network comprising three wireless terminals. BLE has gained traction in various applications due to its advantages of low computational overhead and ease of connection. However, based on the findings presented in Table 10 and Figure 7, our proposed protocol demonstrates a lower computational overhead compared to BLE, with a reduction of 26.4%. This reduction in overhead showcases the efficiency and optimization achieved by our proposed protocol, making it a proper approach for APS environments where power-constrained IoT devices are prevalent.

Furthermore, our proposed protocol offers additional security services, as elaborated in Section 4.3, which BLE fails to provide. These services include perfect forward secrecy, privacy, emergency phase, defense against replay attacks and man-in-the-middle attacks. These security features further enhance the applicability and robustness of our proposed protocol in real-world scenarios.

In conclusion, our analysis reveals that our proposed protocol outperforms BLE in terms of computational overhead and offers advanced security services tailored to the unique requirements of APS environments utilizing power-constrained IoT devices.

## 6. Conclusions

The increasing demand for self-operating APS to elevate the medical care of patients with Type 1 Diabetes has opened a wide range of security threats due to its structure and inevitable integration with wireless transmission medium. Hence, protecting the communication channels of all the devices composing the APS is of paramount importance since sensitive information about the patient is involved. It is essential to balance security, reliability, and resiliency when crafting solutions that ensure patient safety and the protection of personal information. To this end, we designed a security protocol for the APS environment and formally verified its security and correctness using BAN logic and AVISPA. Furthermore, the proposed protocol was also emulated in a controlled environment with actual devices while comparing its computation and communication overhead with other existing work. Based on the experimental results, the proposed protocol demonstrated superior security and performance over existing protocols and standards for the IoT ecosystem.

Healthcare-target systems commonly collect and analyze the health information of patients or users, which can then be utilized for clinical decision support in providing appropriate medical care, preventive measures, and the like. Meanwhile, this article will explore the possibility of leveraging the biological distinction of a patient for authentication strategy, potentially elevating the proposed security protocol.

It would be highly impactful to utilize artificial intelligence and machine learning technology in analyzing medical data and delivering personalized treatment approaches to patients. In our future research, we will design protocols that leverage artificial intelligence to determine patient-specific insulin dosage instead of solely exchanging information on dosage and remaining supply. Additionally, we intend to implement our previously studied attack detection technique [11,12] for APS, along with the proposed protocol, in a real-world setting.

## Figures and Tables

**Table 1:** Notations.

Notation	Meaning
CGM	Continuous Glucose Monitoring
CONT	Controller
IP	Insulin Pump
Sid	Session ID
$PWD_{X}$	X’s passcode
X,Y,Z	ECDHE private key
$g^{X}$, $g^{Y}$, $g^{Z}$	ECDHE public key
$n_{X}$	x-th nonce
$SK_{X}$	Secret key between X and controller
$SK_{EMG}$	Secret key for emergency situation
$HM_{X}$	x-th Hash-based message authentication code
CMD	Command message
dose	Insulin dose
IoB	Insulin that is still active in the body
Heartbeat	Message to check the status of the controller
Response	Message to respond to Heartbeat message
Alert	Message to inform the emergency situation
BGL	Blood glucose level

**Table 2:** Ephemeral option of elliptic curve Diffie–Hellman key exchange.

ECDHE Domain Parameters (*p*, *a*, *b*, *g*, *n*, and *h*) are Pre-Shared between Alice and Bob.
**1. ECDHE Key Generation **
1. Alice generates an ephemeral private key *X* from {1,…,n−1}.
2. Alice derives a public key $g^{X}$ from *X*.
3. Bob also generates own ephemeral private key *Y* from {1,…,n−1}.
4. Bob derives own ECDHE public key $g^{Y}$ from *Y*.
**2. ECDHE Session Key Exchange**
1. Alice sends the public key $g^{X}$ to Bob.
2. Bob computes the ECDHE session key *S* via the following operation.
$S={\left(g^{X}\right)}^{Y}=g^{XY}$
3. After computing the ECDHE session key, Bob sends the public key $g^{Y}$ to Alice.
4. Alice also computes the ECDHE session key *S* via the following operation.
$S={\left(g^{Y}\right)}^{X}=g^{YX}=g^{XY}$
5. Alice and Bob successfully exchange the ECDHE session key *S* and remove each ephemeral private key.
**ECDHE domain parameter**
*p*: a prime number indicating the finite field size;
*a*, *b*: the coefficients for the chosen elliptic curve equation;
*g*: the base point to generate a subgroup;
*n*, *h*: the order and co-factor of the subgroup;

**Table 3:** Notations of BAN logic.

Notation	Meaning
P∣≡X	*P* believes the message *X*
P◃X	*P* receives the message *X*
P∣∼X	*P* previously sent the message *X*
P⇒X	P has authority over *X*
#(X)	The message *X* is fresh
${\langle X\rangle }_{K}$	*X* is combined with a secret *K*
${\left\{X\right\}}_{K}$	*X* is encrypted with a key *K*
$P\overset{K}{↔}Q$	*K* is a secret key shared between *P* and *Q*
$\overset{K}{⟼}P$	*K* is the public key of *P*
$P\overset{K}{⇋}Q$	*K* is a shared secret between *P* and *Q*

**Table 4:** Rules of BAN logic.

Rule	Formula
Message Meaning Rule (MM)	$\frac{P\;|\equiv \;P\;\overset{K}{↔}\;Q,\;P\;◃\;{\left\{X\right\}}_{K}}{P\;|\equiv \;Q\;|∼\;X}$ $\frac{P\;|\equiv \;P\;\overset{K}{⇋}\;Q,\;P\;◃\;{\langle X\rangle }_{K}}{P\;|\equiv \;Q\;|∼\;X}$ $\frac{P\;|\equiv \;\overset{K}{⟼}\;Q,\;P\;◃\;{\left\{X\right\}}_{Q^{-1}}}{P\;|\equiv \;Q\;|∼\;X}$
Nonce Verification Rule (NV)	$\frac{P\;|\equiv \;\#(X),\;P\;|\equiv \;Q\;|∼\;X}{P\;|\equiv Q\;|\equiv \;X}$
Jurisdiction Rule (JR)	$\frac{P\;|\equiv \;Q\;\Rightarrow \;X,\;P\;|\equiv \;Q\;|∼\;X}{P\;|\equiv \;X}$
Freshness Rule (FR)	$\frac{P\;|\equiv \;\#(X)}{P\;|\equiv \;\#(X,\;Y)}$
Decomposition Rule (DR)	$\frac{P\;◃\;(X,\;Y)}{P\;◃\;X}$
Belief Conjunction Rule (BC)	$\frac{P\;|\equiv \;X,\;P\;|\equiv \;Y}{P\;|\equiv \;(X,\;Y)}$ $\frac{P\;|\equiv \;Q\;|\equiv \;(X,\;Y)}{P\;|\equiv \;Q\;|\equiv \;X}$ $\frac{P\;|\equiv \;Q\;|∼\;(X,\;Y)}{P\;|\equiv \;Q\;|∼\;X}$
Diffie-Hellman Rule	$\frac{P\;|\equiv \;Q\;|∼\;\overset{g^{Y}}{⟼}\;Q,\;P\;|\equiv \;\overset{g^{X}}{⟼}\;P}{P\;|\equiv \;P\;\overset{g^{XY}}{↔}\;Q}$ $\frac{P\;|\equiv \;Q\;|∼\;\overset{g^{Y}}{⟼}\;Q,\;P\;|\equiv \;\overset{g^{X}}{⟼}\;P}{P\;|\equiv \;P\;\overset{g^{XY}}{⇆}\;Q}$

**Table 5:** Comparison table of security requirements and goal achievements.

Security Requirements	Goals	Derivation
Mutual Authentication	CONT – CGM: (22), (25) CONT – IP: (29), (32) CGM – IP: (42), (43)	CONT – CGM: (47), (54) CONT – IP: (62), (69) CGM – IP: (93), (97)
Secure Key Exchange	$SK_{CGM}$: (24), (26), (28), (36) $SK_{IP}$: (31), (33), (35), (39)	$SK_{CGM}$: (50), (55), (58), (77) $SK_{IP}$: (65), (70), (73), (85)
Perfect Forward Secrecy	CONT – CGM: (23), (27) CONT – IP: (30), (34) CGM – IP: (37), (40)	CONT – CGM: (49), (57) CONT – IP: (64), (72) CGM – IP: (79), (87)
Emergency Phase	$SK_{EMG}$: (38), (41), (42), (43)	$SK_{EMG}$: (80), (88), (93), (97)

**Table 6:** Comparison in terms of security requirements with existing protocols.

Protocol	Security Requirements								
	AUTH	CONF	INT	SKE	PFS	PRV	EMEG	RPA	MiTM
Wu et al. [13]	◯	◯	◯	◯	×	×	×	◯	◯
Chi et al. [14]	◯	◯	◯	◯	×	×	×	◯	◯
HAT [15]	◯	◯	◯	◯	◯	×	×	◯	◯
Bluetooth LE [16]	◯	◯	◯	◯	×	×	×	×	×
TLS 1.3 [18]	◯	◯	◯	◯	◯	◯	×	◯	◯
Our Proposed	◯	◯	◯	◯	◯	◯	◯	◯	◯

◯: Support; ×: Not support; AUTH: Mutual Authentication; CONF: Confidentiality; INT: Integrity; SKE: Secure Key Exchange; PFS: Perfect Forward Secrecy; PRV: Privacy; EMEG: Emergency phase; RPA: Defense against replay attack; MiTM: Defense against man-in-the-middle attack.

**Table 7:** Comparison in terms of computational overheads with existing protocols.

Protocol	Overhead
Wu et. al. [13]	$10T_{H}+3T_{SE}+3T_{SD}+1T_{AE}+1T_{AD}+2T_{SIGN}+2T_{VER}$
Chi et. al. [14]	$19T_{H}+4T_{SE}+4T_{SD}+1T_{AE}+1T_{AD}+2T_{SIGN}+2T_{VER}$
HAT [15]	$9T_{H}+2T_{SE}+2T_{SD}+2T_{DHE}$
Bluetooth LE * [16]	$2(12T_{H}+2T_{DHE})$
TLS 1.3 * [18]	$2(22T_{H}+1T_{SIGN}+1T_{VER}+2T_{DHE})$
Our Proposed	$16T_{H}+6T_{DHE}$

$T_{H}$: Cryptographic hash function; $T_{SE}$: Symmetric encryption; $T_{SD}$: Symmetric decryption; $T_{AE}$: Asymmetric encryption; $T_{AD}$: Asymmetric decryption; $T_{SIGN}$: Digital signature; $T_{VER}$: Digital signature verification; $T_{DHE}$: Elliptic curve Diffie–Hellman Ephemeral; * To facilitate comparison in this paper, protocols generally used in two-party environments were; reconfigured into three-party settings for APS environments.

**Table 8:** Comparison in terms of communication overheads with existing protocols.

Protocol	Round-Trip Times (RTT)
Wu et. al. [13]	3
Chi et. al. [14]	6
HAT [15]	1
Bluetooth LE [16]	4
TLS 1.3 [18]	1
Our Proposed	3

**Table 9:** Test environment specification.

Specification	Arduino Nano 33 (Insulin Pump)	Galaxy S 20 (Android Controller)	nRF52840 (CGM)
CPU Core	32-bit ARM^®^ Cortex^^®^^-M4	Samsung Exynos 990(Exynos M5 MP2 + ARM^®^ Cortex^®^ - A76 MP2 + ARM^®^ Cortex^®^ - A55 MP4)	32-bit ARM^®^ Cortex^®^-M4
Ram	64 KB Flash ram 512 KB Sram	8 GB	64 KB Flash ram 512 KB Sram
Voltage	3.3 V	3.85–4.4 V	3.3 V
Programming Language	C++	Kotlin	C

**Table 10:** Comparison results of computation overhead.

	Arduino Nano 33 (Insulin Pump)	Galaxy S 20 (Android Controller)	nRF52840 (CGM)
Proposed protocol	333.6 ms	0.76 ms	68.24 ms
Bluetooth LE	452.8 ms	1.28 ms	91.44 ms

## Data Availability

Not applicable.
